# Chloride Intracellular Channel Protein 1 (CLIC1) Is a Critical Host Cellular Factor for Influenza A Virus Replication

**DOI:** 10.3390/v16010129

**Published:** 2024-01-16

**Authors:** Mahamud-ur Rashid, Kevin M. Coombs

**Affiliations:** 1 Department of Medical Microbiology and Infectious Diseases, University of Manitoba, Room 543 Basic Medical Sciences Building, 745 Bannatyne Avenue, Winnipeg, MB R3E OJ9, Canada; 2 Manitoba Centre for Proteomics and Systems Biology, Room 799, 715 McDermot Avenue, Winnipeg, MB R3E 3P4, Canada; 3 Children’s Hospital Research Institute of Manitoba, Room 513, John Buhler Research Centre, 715 McDermot Avenue, Winnipeg, MB R3E 3P4, Canada

**Keywords:** influenza A virus, CLIC1, host factor, transcription, NPPB, replication cycle

## Abstract

(1) Background: Influenza A Virus (IAV) uses host cellular proteins during replication in host cells. IAV infection causes elevated expression of chloride intracellular channel protein 1 (CLIC1) in lung epithelial cells, but the importance of this protein in IAV replication is unknown. (2) In this study, we determined the role of CLIC1 in IAV replication by investigating the effects of CLIC1 knockdown (KD) on IAV viral protein translation, genomic RNA transcription, and host cellular proteome dysregulation. (3) Results: CLIC1 KD in A549 human lung epithelial cells resulted in a significant decrease in progeny supernatant IAV, but virus protein expression was unaffected. However, a significantly larger number of viral RNAs accumulated in CLIC1 KD cells. Treatment with a CLIC1 inhibitor also caused a significant reduction in IAV replication, suggesting that CLIC1 is an important host factor in IAV replication. SomaScan^®^, which measures 1322 proteins, identified IAV-induced dysregulated proteins in wild-type cells and in CLIC1 KD cells. The expression of 116 and 149 proteins was significantly altered in wild-type and in CLIC1 KD cells, respectively. A large number of the dysregulated proteins in CLIC1 KD cells were associated with cellular transcription and predicted to be inhibited during IAV replication. (4) Conclusions: This study suggests that CLIC1 is involved in later stages of IAV replication. Further investigation should clarify mechanism(s) for the development of anti-IAV drugs targeting CLIC1 protein.

## 1. Introduction

Seasonal flu caused by influenza A virus (IAV) infects about one billion people worldwide every year, resulting in 3 to 5 million cases of severe sickness and roughly 500,000 deaths [1]. In addition, pandemic IAV events cause millions of deaths. During the previous century, IAV likely caused about 100 million fatalities worldwide [2,3]. So far, 18 hemagglutinin (HA) and 11 neuraminidase (NA) IAV subtypes have been described, based on antigenic variations [4,5]. H1N1 and H3N2 strains are the most common influenza viruses responsible for seasonal human influenza infections [1].

The IAV genome consists of eight negative-sense single-stranded RNA segments [4]. Because of the highly mutation-prone genome, the virus changes frequently, develops resistance to antiviral drugs, and escapes neutralizing antibodies even after vaccination [6]. As a result, developing an effective vaccine against IAV is very difficult, and treatment options become limited. However, as intracellular parasites, viruses rely on host factors to complete their replication and evade immune responses. Thus, intracellular virus replication could be inhibited by disrupting a host protein activity or signaling pathway required for virus replication. Therefore, it is necessary to identify host factors critical for IAV replication and to clearly comprehend their role(s) in IAV replication.

The chloride intracellular channel (CLIC) protein 1 was more highly expressed in A549 cells after highly pathogenic IAV-H5N1 infection [7] and siRNA knockdown (KD) screening showed a significant reduction in IAV-PR8 replication after CLIC1 KD [8]. These observations suggest that CLIC1 could be a critical host factor for IAV replication. The CLICs are widely distributed in the endoplasmic reticulum, mitochondria, and nuclear membranes. Different CLICs may localize in various sites of cells or tissue, and they exhibit similar functional properties due to the extensive homologies in their amino acid sequences [9,10]. Among the other CLIC family members, CLIC1 is one of the most studied proteins and was first discovered in humans [10,11]. CLIC1 is a 27-kDa monomer protein that serves as an intracellular chloride channel and is found in both soluble and integral membrane states in the nucleus and cytoplasm [12,13]. The roles of this protein include maintaining cell membrane potential and cell volume, transport of molecules, intracellular pH regulation, etc. The tetrameric configuration of the subunits in CLIC1 is sufficient to create a functional ion channel [14,15].

CLIC1 is expressed in various cell types and is most prevalent in the skeletal muscle and heart cells [13]. Higher expression of CLIC1 was detected in different types of cancer cells [16]. As a chloride channel, CLIC1 can transform cells by increasing cell proliferation, migration, and invasiveness [10]. Interestingly, Merkel cell polyomavirus (an oncogenic virus) can also induce cell transformation with the help of CLIC1 [17]. Knockdown of CLIC1 protein resulted in a significant reduction of West Nile virus [18] and vaccinia virus [19]. However, it is unclear how CLIC1 functions in the IAV life cycle. In this study, we identified the role of CLIC1 in IAV replication by investigating the effects of CLIC1 KD on IAV viral protein translation, genomic RNA transcription, and host cellular proteome dysregulation.

## 2. Materials and Methods

### 2.1. Cells and Viruses

Human A549 lung carcinoma epithelial cells and Madin–Darby canine kidney (MDCK) cells were grown in DMEM medium (GIBCO, Grand Island, NY, USA, Cat. 21013024) supplemented with sodium-pyruvate, non-essential amino acids (NEAA) and l-glutamine. For A549 and MDCK cells, 10% and 5% Fetal Bovine Serum (FBS) (Thermo Fisher Scientific, Waltham, CA, USA, Cat A4766801) was used in the media, respectively. A detailed protocol was published previously [20]. Human fetal lung cells (MRC-5; ATCC, Manassas, VA, USA, Cat # CCL-171) were purchased from ATCC and grown in complete EMEM (ATCC, Manassas, VA, USA, Cat # 30-2003) media containing 10% FBS. All three cell lines were maintained at 37 °C in 5% CO_2_ and passaged by trypsinization three times each week to keep the cells growing in monolayer. Four strains of IAV were used in this study; A/New Caledonia/20/1999 (H1N1; N-Cal), A/WSN/1933 (H1N1; WSN), A/Mexico/INDRE4487/2009 (H1N1; pdm09), and A/PR/8/34 (H1N1; PR8). The virus stocks were prepared by infecting MDCK cells at an MOI of 0.01 plaque-forming units (PFU)/cell and supernatants were collected at 45 h post infection (hpi). The supernatants were centrifuged at 64,000× *g* for 2 h at 4 °C to concentrate the virus stocks. Finally, the concentrated pellets were resuspended in phosphate buffered saline (PBS) containing 10% glycerol and stored at −80 °C until used.

### 2.2. Infection and Plaque Assay

A549 cells were grown in 12-well plates in complete DMEM medium. At 70–80% confluency, the cells were washed twice with 1× PBS to remove the FBS and infected with PR8, pdm09 or WSN strains at MOI = 0.01. Supernatants were collected at 0, 2, 4, 8,16, 24, 36, and 45 hpi from non-silenced (scrambled siRNA control) wildtype and from CLIC1 KD cells. The numbers of infectious virus particles in the supernatant samples were determined by plaque assays. In brief, the supernatants were diluted serially 1:10 in gel saline to 10^7^ dilutions. The samples from each dilution (100 µL) were inoculated onto monolayers of MDCK cells in duplicate into different wells of 6-well/12 well plates. After one hour of adsorption, the infected plates were overlayed with 1× DMEM medium, containing 0.8% Avicel, 0% FBS, and 2.5 μg/mL trypsin. To prevent bacterial contamination, the overlays were supplemented with antibiotics (gentamicin and amphotericin B). After 72 h of incubation at 35 °C the overlay media were removed, and plates were washed with 1× PBS. The MDCK cells were fixed using 2.5% formaldehyde for 1 h and stained with crystal violet for another 1 h. The stain was rinsed, and the plates were dried at room temperature. The number of plaques were counted and back-calculated to determine PFU/mL [20].

### 2.3. Cell Viability

The effect of CLIC1 KD on cell viability was assessed using the WST-1 (Roche, Basel, Switzerland) reagent as directed by the manufacturer. Eighth thousand A549 cells were added to each well of a 96-well plate, and, after 24 h of incubation at 37 °C, cells were treated with non-silencing (NSC) scrambled, or CLIC1, siRNAs. To determine the cell viability at 48- or 72-h post-transfection (hpt) in each well, 9 μL WST-1 reagent was added. The 96 well plates were incubated for 2 h at 37 °C. The colorimetric variations in the media were measured with a photo-densitometer and were used to calculate cell viability. Cell viability was calculated by comparing them to time-matched NSC treated cells. The experiment was performed in three biological replicates and each biological replicate was averaged from five technical replicates.

### 2.4. siRNA Transfection

The expression of CLIC1 protein was knocked down by siRNA treatment following the protocol described in [21]. In summary, A549 cells were grown in complete DMEM medium, and siRNA transfection was conducted at 30–40% cell confluency. Before transfection, the FBS was removed from the cells by washing two times with RNase-free PBS. As per Dharmacon’s directions, on-target (OT) and smart-pool (SP) siRNAs for CLIC1 (50 nM) and scrambled non-silencing control (NSC) siRNA (50 nM) and Dharmafect (GE Healthcare Dharmacon, Lafayette, CO, USA, Cat. T-2001) were diluted in Opti-MEM medium. After mixing the diluted siRNA and Dharmafect for 20 min at room temperature, they were introduced directly onto the A549 cells in the culture plates. Culture plates were then incubated in 5% CO_2_ at 37 °C. To measure the effect of CLIC1 KD on viral protein expression and RNA replication, cells were infected with IAV at 48 hpt and cells were harvested at different time points up to 45 hpi. To investigate the impact of CLIC1 KD on progeny infectious virus replication, supernatants were collected at different time intervals up to 45 hpi.

### 2.5. Protein Extraction and Quantification 

A549 cells in 6-well plates or 60 mm dishes were transfected with siRNAs and infected with IAV at MOI = 3 PFU/cell. At various time intervals, virus- and mock-infected cells were scraped from culture plates. The cells were washed three times in ice-cold PBS before being lysed by sonication in 60 µL mammalian protein extraction reagent (M-PER, Thermo Fisher Scientific, Waltham, CA, USA, Cat. 78501) detergent containing 1 HALT^®^ Protease inhibitor (Thermo Fisher Scientific, Cat. 78430,). The cell lysates were centrifuged at 14,000× *g* for 10 min at 4 °C to remove the insoluble cellular components, and protein quantities were determined by BCA^TM^ Protein Assay (Pierce; Rockford, IL, USA, Cat. 23225), quantified using bovine serum albumin standards (Thermo Fisher Scientific, Cat. 23208).

### 2.6. SomaScan Analyses

To better understand the role of CLIC1 KD on IAV infection, IAV-induced proteomic dysregulation in non-silenced, scrambled siRNA wild-type cells and in CLIC1 KD cells were assessed and compared after bioinformatic analysis. For this, cell lysates were obtained from triplicate replicates of four different conditions (NSC, NSC + PR8, CLIC1 KD, and CLIC1 KD + PR8 cells) at 24 hpi and expression of cellular proteomes were analyzed using the SomaScan version 1.3K platform, which can concurrently assess 1307 proteins in up to 92 samples. Each biologic sample was mixed with the unique SOMAmers, which recognize and bind to a particular human protein with high specificity [22,23]. The SOMAmers were washed, released, hybridized to DNA microarrays, and the expression values of each targeted protein were quantified in relative fluorescent units (RFU) [23,24]. A standard curve created for each protein-SOMAmer combination confirmed that the RFU levels of each protein expression were directly proportional to the quantities of target proteins in the original samples. RFU were converted to log_2_ and analyzed as described previously [25].

### 2.7. Immunoblotting

Western blot was used to determine viral and host cellular protein expression, following the protocol reported previously [20]. Thirty ug of proteins from different conditions were resolved in 10 or 12% SDS-PAGE gels, then transferred to 0.2 µm nitrocellulose membranes and probed for specific proteins with the following antibodies: anti-PSMA2 (Cell Signaling, Danvers, MA, USA, Cat. 2455), anti-STAT3 (Cell Signaling; Cat #no. 9139S), anti-STAT1 (Cell Signaling, Cat. 9176S), anti-GAPDH (Cell Signaling, Cat.2118L;), anti-Beta-Actin (Cell Signaling, Cat. 3700S), CLIC1anti-CLIC1 (EMD Millipore, Darmstadt, Germany, Cat. MABN46), and in-house prepared IAV mouse-anti-NS1 and mouse-anti-NP [26]. To identify immunological complexes, appropriate secondary horseradish peroxidase (HRP)-conjugated horse anti-mouse or anti-rabbit antibodies (Cell Signaling, cat. 7076, cat. 7074, respectively) were used. Protein bands were visualized with ECL reagents and photographed using an Alpha Innotech FluorChemQ MultiImage III instrument (ALT American Laboratory trading, San Diego, CA, USA). Image J 1.50i was used to measure band intensities to evaluate the differences in protein expression (NIH, Bethesda, Maryland, USA). GraphPad Prism v 9.1.0 (La Jolla, CA, USA) software was used to analyze and visualize the data.

### 2.8. RNA Extraction and Real-Time PCR

To investigate the effect of CLIC1 KD on vRNA transcription, A549 cells were infected with IAV-PR8 at MOI 3 and cells were collected at 24 hpi. Then the cells were washed with cold PBS, and total cellular RNA was extracted using the RNeasy Mini Kit (QIAGEN, Germantown, MD, USA, Cat. 74104). The Go Script^TM^ Reverse Transcription System kit (Promega, Madison, WI, USA, Cat. A5000) was used to generate cDNA from 250 ng of purified mRNA. The qRT-PCR was carried out with the Platinum^TM^ SYBR^TM^ Green qPCR SuperMix-UDG kit (Thermo Fisher, Waltham, CA, USA, Cat. 11733046). The total volume of the master mix was 25 μL, which included 12.5 μL Platinum^TM^ SYBR^TM^ Green qPCR SuperMix (2×), 0.5 μL ROX Reference Dye, 0.5 μL each of the 10 μM forward and reverse primers specified below, 6 μL H_2_O, and 5 μL (10 ng) template cDNA. For each sample, the PCR was carried out in three biological replicates and two technical duplicates. The QuantStudio^TM^ 3 Real-Time PCR System was used for all PCR experiments (Applied Biosystems, Waltham, MT, USA). The PCR cycle conditions were 50 °C for 2 min, 95 °C for 2 min, and 40 cycles of 95 °C for 15 s and 60 °C for 30 s. Ct values were normalized to 18S rRNA controls before being compared to non-silencing siRNA controls. The primer sequences were PR8-HA (Fwd:CATTCCGTCCATTCAATCC; Rev: AACCATACCATCCATCTATC), PR8-NP (Fwd: AGAGGGTCGGTTGCTCACAA; Rev: TGGCTACGGCAGGTCCATA), and PR8-NS1 (Fwd: CTTCGCCGAGATCAGAAATC; Rev: TGGACCATTCCCTTGACATT).

### 2.9. Impact of CLIC1 Inhibitors on IAV Replication

The effects of 5-nitro-2-(3-phenylpropyl-amino) benzoic acid (NPPB), a CLIC1 inhibitor (Santa Cruz Biotechnology, Dallas, TX, USA, Cat. sc-201542), on IAV replication were evaluated. A549 and MRC-5 cells were treated for 48 h in serum-free medium with several concentrations of NPPB (0–250 µM) to find the highest concentration of the drug with low cytotoxicity (>80% cell viability). The WST-1 test was used to assess the drug’s cytotoxic effect. After 48 h of treatment, 62.5 µM or lower concentrations of NPPB showed cell viability of more than 80% in A549 cells. To test the impact of the drugs on IAV replication, 62.5 µM concentrations were tested. The A549 cells were first pre-treated with the drugs for 2 h before being infected with IAV PR8 or N-Cal strains. After one hour of virus adsorption, the cells were overlayed with serum-free DMEM media, containing the same concentrations of the drug and incubated at 37 °C. The overlay media were also supplemented with antibiotics (100 μg/mL gentamicin and 100 μg/mL amphotericin B) and 2.5 μg/mL trypsin. At 45 hpi, virus samples from the supernatants were collected and titrated by plaque assay.

### 2.10. Photomicrography

A549 cells were photographed at 200× magnification with a CanonA700 digital camera (Canon, Ota City, Tokyo, Japan) to see the effect of siRNA transfection at 4 dpt. Images were imported into Microsoft PowerPoint (Microsoft, Redmond, WA, USA) and minor brightness and contrast adjustments were performed that did not change the image context in relation to each other.

### 2.11. Immunofluorescent Microscopy 

Approximately 4000 A549 cells were seeded into each well of a 6 mm Multi-Spot Slide (Fisher Scientific, Waltham, MT, USA, Cat. 99-910-90) and grown for 24 h at 37 °C in 10% FBS supplemented DMEM medium. The cells were subsequently treated with either 50 nM CLIC1 or 50 nM NSC siRNAs for 48 h and infected with IAV-PR8 at MOI 3. At 24 hpi, each spot was rinsed five times with 1× PBS and fixed with 4% paraformaldehyde for 15 min before being washed five times with 1× PBS and permeabilized with 0.1% Triton X-100 for five min. The fixed cells were then blocked overnight at 4 °C with 3% bovine serum albumin (BSA). Cells were then treated overnight in 3% BSA at 4 °C with primary anti-CLIC1 antibodies or in-house produced IAV mouse anti-NS1 [26]. Cells were then rinsed five times with 1× PBS and treated with 0.2% Tween 20 (PBT) and an anti-rabbit secondary antibody that was labeled with Alexa Fluor488 for 60 min. Finally, DAPI mounting dye was applied to every spot of the slide. A Zeiss Axio Observer Z1 inverted fluorescence microscope was used to observe the fluorescent images. 

### 2.12. Statistical and Bioinformatics Analyses

The RFU value of each protein from SomaScan^®^ was converted to Log_2_ values. Then the delta Log_2_ value was calculated by subtracting the Log_2_ expression value of each PR8-infected protein from each corresponding mock-infected Log_2_ expression value. The delta Log_2_ values were further transformed into fold-change values. The significance of the expression change was determined by Students’ *t*-test (2 tails) and Z-score analyses based on the three replicates of protein expression. For further bioinformatic analysis with Ingenuity Pathway Analysis (IPA) software (Version: v01-22-01) proteins with a significant dysregulation in expression (*p*-value < 0.05 or Z-score values of ≥+1.96σ and ≤−1.96σ) and fold change >±1.3 were selected. The band intensities of Western-blot images were measured using ImageJ 1.51K software (NIH, USA). The quantitative values of Western-blot images were analyzed for statistical analysis using one-way or two-way ANOVA in GraphPad Prism 6.0 (*p*-values < 0.05). Heatmaps were created using the online free tool MORPHEUS (Broad Institute, Cambridge, MA, USA).

## 3. Results

### 3.1. Optimization of CLIC1 Knockdown by siRNA Treatment

**Figure 1:**
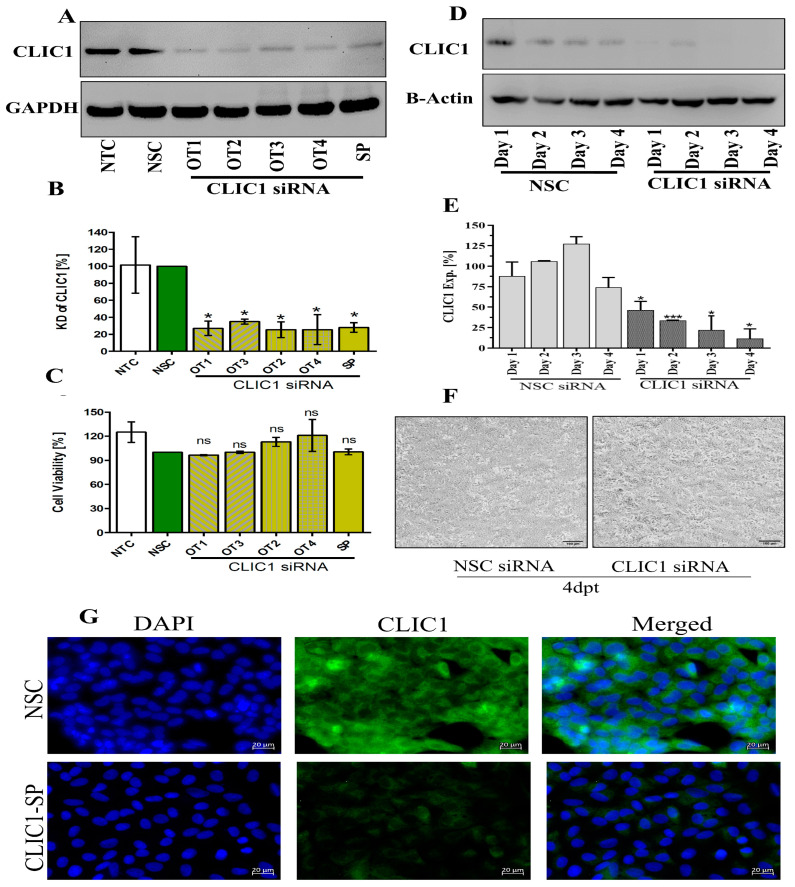
Optimization of CLIC1 knockdown by siRNA transfection in A549 cells. (**A**) Expression of CLIC1 protein was detected by Western blot after 48 h of treatment with 50 nM OT and SP CLIC1 siRNA. (**B**) Quantitative expression of CLIC1 from Western blots after OT and SP CLIC1 siRNA treatment. (**C**) Cell viability of A549 cells after 48 h of siRNA transfection. (**D**) Expression of CLIC1 detected by Western blot up to 4 days after 50 nM SP CLIC1 siRNA treatment. (**E**) Quantitative expression of CLIC1 from Western blot images after treatment with 50 nM CLIC1 siRNA up to 4 dpt. (**F**) Photomicrographs of A549 cells showing the impact of siRNA treatment on A549 cell phenotypes at 4 dpt with 50 nM CLIC1 siRNA treatment; scale bars are 100 µm. (**G**) CLIC1 KD was confirmed in CLIC1 siRNA-treated cells by immunofluorescence microscopy (scale bar is 20 µm). DAPI was used to visualize the cell nuclei. ns = Not significant. *: *p* < 0.05, ***: *p* < 0.001.

To KD the expression of CLIC1, A549 cells were transfected with 50 nM of four OT and SP siRNA targeted against CLIC1 for 48 h. All the OT and SP siRNA caused a significant reduction of CLIC1 proteins (Figure 1A,B) without any significant impact on cell viability (Figure 1C). The A549 cells were further treated with 50 nM CLIC1 SP siRNA for four days to determine the KD stability over time. On days 1, 2, 3, and 4, CLIC1 expressions were reduced to 46%, 33%, 21%, and 11%, respectively (Figure 1D,E). After four days of transfection, the morphology of A549 cells was not visually different between NSC and CLIC1 siRNA treatment (Figure 1F). CLIC1 protein knockdown was further confirmed by immunofluorescence microscopy (Figure 1G).

### 3.2. Impact of CLIC1 KD on IAV Replication

**Figure 2:**
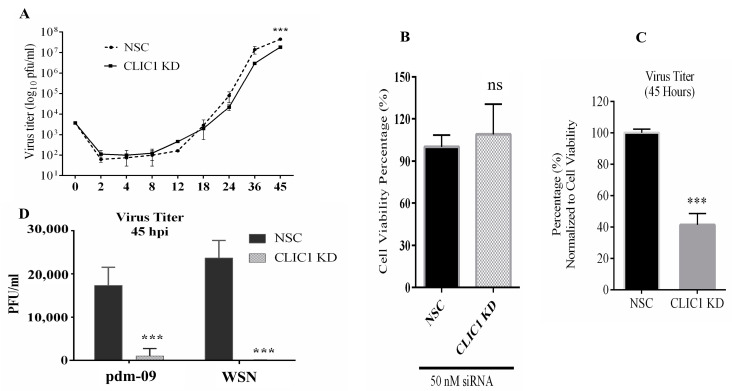
CLIC1 KD impairs IAV replication. After 48 h of either NSC or CLIC1 siRNA (CLIC1 KD) treatment, A549 cells were infected with IAV-PR8 at MOI 0.01. At 0, 2, 4, 8, 12, 18, 24, 36, and 45 hpi, supernatant from the infected cells were collected. Similarly, pdm-09 and WSN strains were used to infect NSC and CLIC1 KD cells, and supernatants were collected at 45 hpi. Plaque assay was used to assess the viral titers. (**A**) PR8 titers in the CLIC1 KD cells’ supernatant over time in comparison to NSC. (**B**) WST-1 assay was used to evaluate cell viability 96 h after siRNA transfection. (**C**) The percentage of viral titer in the supernatant from CLIC1 KD cells at 45 hpi when compared to the control and normalized to cell viability. (**D**) pdm09 and WSN titers in NSC- and CLIC1 KD cells. Replicates: *n* = 3; ns = Not significant, ***: *p* < 0.001.

The effect of CLIC1 KD on progeny viral replication was evaluated. To do that, CLIC1 protein expression was knocked-down in A549 cells by siRNA treatment and cells were then infected with IAV-PR8. The supernatants were collected at various time points up to 45 hpi, and infectious progeny virus titers determined by plaque assay. At 45 hpi, CLIC1 KD significantly reduced the number of progeny viruses in the supernatant (Figure 2A). However, CLIC1 KD had no apparent effect on cell viability (Figure 2B). When the viral titer in the supernatant was normalized to cell viability, virus titer in CLIC1 KD cells was reduced about 60% (Figure 2C). Not only did CLIC1 KD have an effect on the PR8 strain, but it also significantly decreased the replication of the pdm-09 and WSN viruses (Figure 2D).

### 3.3. The Chloride Channel Inhibitor NPPB Suppresses the Replication of IAV Virus

**Figure 3:**
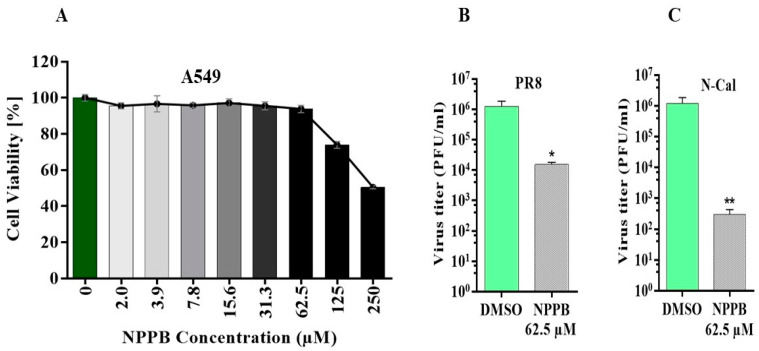
Chloride channel inhibitor NPPB suppresses the replication of IAV. (**A**) The cytotoxicity of NPPB was determined by treating A549 cells with different concentrations of the drug and cell viability was measured by WST-1 assay. To determine the impact of the NPPB drug on virus replication, A549 cells were pre-treated with the drug and infected with IAV PR8 and N-Cal strains at MOI = 0.01. The drug was also added to the overlay media after infection. PR8 and N-Cal were collected at 45 hpi. Impact of NPPB on (**B**) PR8 and (**C**) N-Cal replication in A549 cells. The light or dark green colour bar represents the DMSO control, and the lower to higher darker grayscale gradient bars represent the low to a high concentration of NPPB. Replicates: *n* = 3. *: *p* <0.05, **: *p* < 0.01.

Knockdown of CLIC1 expression caused a significant impact on IAV replication, indicating that CLIC1 could be a critical host factor for IAV replication. Thus, we examined whether the chloride channel inhibitor 5-nitro-2-(3-phenylpropyl-amino) benzoic acid (NPPB) could also impact IAV replication. The toxicity of the different concentrations of the drug was tested in A549 (Figure 3A) cells. Based on the drug’s cytotoxicity, 62.5 μM concentrations of NPPB were used in A549 cells to determine the impact of the drug on IAV replication. NPPB significantly reduced the replication of IAV strains PR8 (Figure 3B) and N-Cal (Figure 3C) in A549 cells.

### 3.4. Impact of CLIC1 KD on Viral Protein and RNA Expression

**Figure 4:**
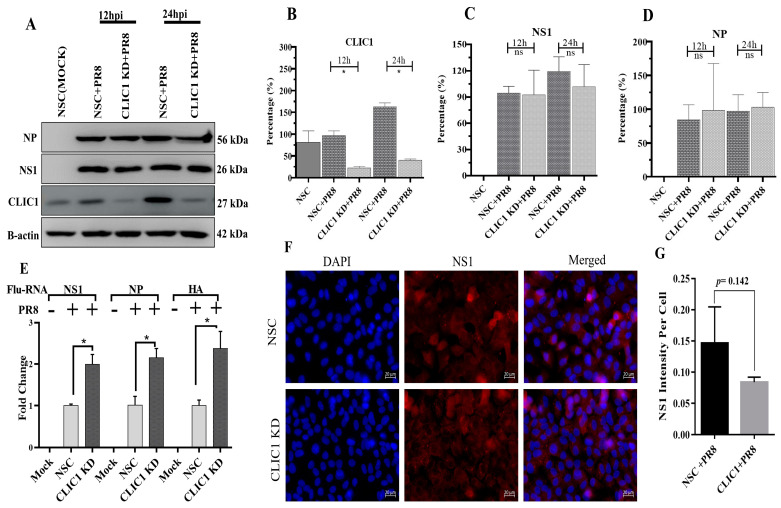
Impact of CLIC1 KD on IAV viral protein translation and viral RNA transcription. After 48 h of either NSC or CLIC1 siRNA (CLIC1 KD) treatment, A549 cells were infected with IAV-PR8 at MOI 3. For Western blot analyses of the expression of viral proteins, cell lysates from PR8-infected cells were extracted at 12 and 24 hpi. After 24 hpi, cells were fixed on slides for immune fluorescence microscopy observations of viral protein localization. Viral RNAs were also extracted at 24 hpi, and RT-qPCR was performed to identify the relative viral RNA transcript numbers. (**A**) Western blot analyses of IAV-PR8 NP and NS1 protein expression in CLIC1 cells at 12 and 24 hpi. (**B**) CLIC1 expression, (**C**) IAV-NS1 expression, and (**D**) IAV-NP protein expression. (**E**) Comparison of IAV-NS1, NP, and HA vRNA transcripts in mock-infected (NSC control) cells to those in CLIC1 KD cells. (**F**) Immunofluorescence images demonstrating the expression of the NS1 protein in CLIC1 KD cells that were infected with IAV-PR8. (**G**) Total intensity of NS1 was measured and divided by number of nuclei to determine the per cell intensity. h = hours, ns = Not significant. *: *p* < 0.05.

To identify the specific step(s) in IAV replication that CLIC1 KD impacted since IAV progeny virus production was drastically reduced in CLIC1 KD cells, we started by evaluating the effect on viral protein translation. CLIC1 KD and NSC A549 cells were infected with PR8 at MOI = 3. At 12, 24, 36, and 48 hpi, infected cells were collected. Western blot analyses of cell lysates were done to detect the expression profiles of IAV-NS1, IAV-NP, and CLIC1 proteins (Figure 4A). Quantification of band intensities from Western blots (Figure 4B) confirmed a significant decrease in CLIC1 expression. The CLIC1 KD had no significant effect on viral protein expression (Figure 4C,D). Then, we investigated the effect of CLIC1 KD on total viral RNAs by using RT-qPCR. RNA was obtained from NSC and CLIC1 KD cells at 24 hpi after PR8 infection at MOI = 3. Reverse transcription was used to create the cDNA, and qPCR was run using viral NS1, NP, and HA RNAs as the targets. Total NS1, NP, and HA RNAs were significantly higher in CLIC1 KD cells (Figure 4E). The effect of CLIC1 KD on the localization of viral proteins was further examined by infecting CLIC1 KD and NSC cells at MOI = 3 and fixing the cells 24 h later. IAV-NS1 protein was visualized using immune fluorescence microscopy in NSC and CLIC1 KD cells after IAV infection. Interestingly, the IAV-NS1 protein intensity was not significantly affected by IAV infection in CLIC1 KD cells compared to the NSC cells (Figure 4F,G).

### 3.5. Proteomic Dysregulation Caused by CLIC1 KD during IAV Infection

We subsequently assessed the effects of PR8 infection on CLIC1 KD cells by detecting dysregulation of the cellular proteome in order to understand the function of CLIC1 in the IAV replication process. We used SomaScan, which can carry out quantitative assessments of 1307 proteins concurrently from up to 92 samples, in order to identify cellular proteome dysregulation [23]. The implications of PR8 infection on wild type and CLIC1 KD A549 cellular proteomes were assessed by contrasting the cellular proteomes of PR8-infected vs. NSC (NSC + PR8) and CLIC1 KD + PR8 vs. CLIC1 KD cells (CLIC1 KD + PR8), respectively. There was a significant dysregulation of 218 and 352 proteins because of PR8 infection and CLIC1 KD + PR8 infection, respectively (Table 1). 

However, using threshold values of ≥+1.3 or ≤−1.3-fold change and *p* values of <0.05 resulted in identification of 116 (31 up-regulated and 85 down-regulated) proteins from PR8 infection, and 149 (22 up-regulated and 127 down-regulated) proteins from CLIC1 KD + PR8 infection that were considered for bioinformatics analysis. The list of proteins dysregulated ≥1.5-fold in either direction is listed in Table 2. Western blots were performed to validate the expression of cellular proteins STAT1, STAT3, PSMA2, and CLIC1 (Supplementary Figure S2). 

**Figure 5:**
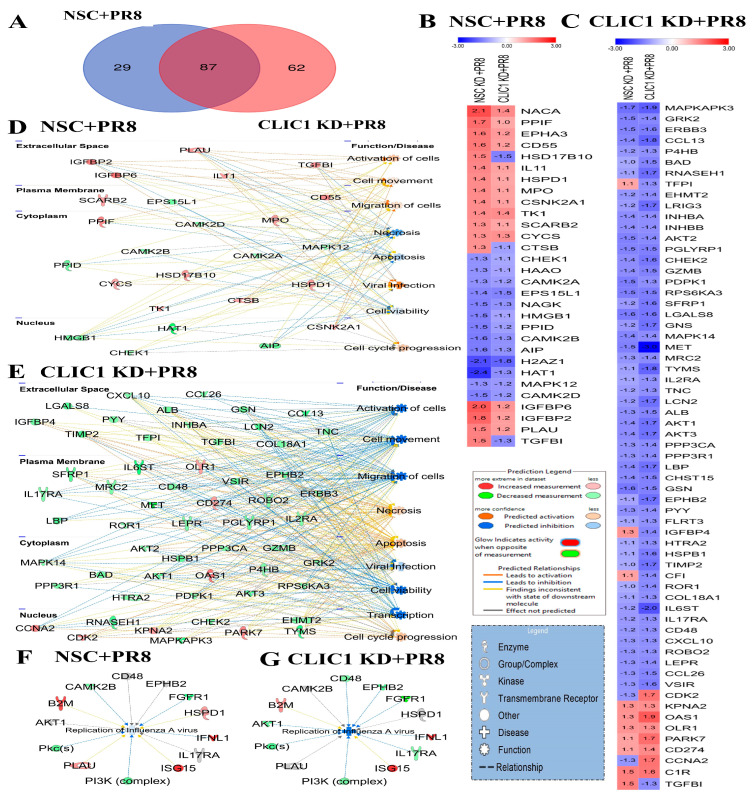
Impact of CLIC1 KD on the A549 cellular proteome during IAV infection. (**A**) Venn diagram showing the numbers of proteins dysregulated in non-silencing control (NSC + PR8) and CLIC1 KD cell (CLIC1 KD + PR8) after PR8 infection. (**B**) Heatmap of the proteins significantly dysregulated only in NSC + PR8 cells but not significantly impacted in CLIC1 KD + PR8 cells. (**C**) Heatmap of the proteins significantly dysregulated only in CLIC1 KD + PR8 cells but not significantly impacted in NSC + PR8 cells. Red: up-regulated; Blue: down-regulated. The numbers inside the boxes indicate the fold change expression values of the proteins. (**D**) Association of the proteins dysregulated only in NSC + PR8 cells with cellular functions. (**E**) Association of the proteins dysregulated only in CLIC1 KD + PR8 cells with cellular functions. IPA predicted the impact of dysregulated protein expression on IAV replication in (**F**) NSC + PR8 and (**G**) CLIC1 KD + PR8 cells.

There were 29 proteins significantly altered in wild-type cells but not significantly affected in CLIC1 KD cells after PR8 infection (Figure 5A,B), while 62 proteins were significantly altered in CLIC1 KD + PR8-infected cells but not significantly changed by PR8 infection in wild type cells (Figure 5A,C). However, transforming growth factor-beta 1 (TGFB1) was the only protein significantly up-regulated in wild-type cells but significantly down-regulated in CLIC1 KD cells after PR8 infection. Bioinformatic analysis of the differentially regulated protein in NSC + PR8 and CLIC1 KD + PR8 cells by Ingenuity Pathway Analysis (IPA) revealed that activation of cells, cell movement, migration of cells, cell cycle progression, and viral infection were activated/increased by PR8 infection in wild-type cells but inhibited/decreased in CLIC1 KD cells. Where necrosis and apoptosis were inhibited by IAV infection in wild type cells, they were activated in CLIC1 KD cells after PR8 infection (Figure 5D,E). Several of these differentially regulated proteins were associated with IAV replication. Based on the expression values, IPA could not anticipate any significant impact on IAV replication in wild-type cells, but IAV replication was predicted to be inhibited in CLIC1 KD A549 cells (Figure 5F,G).

**Figure 6:**
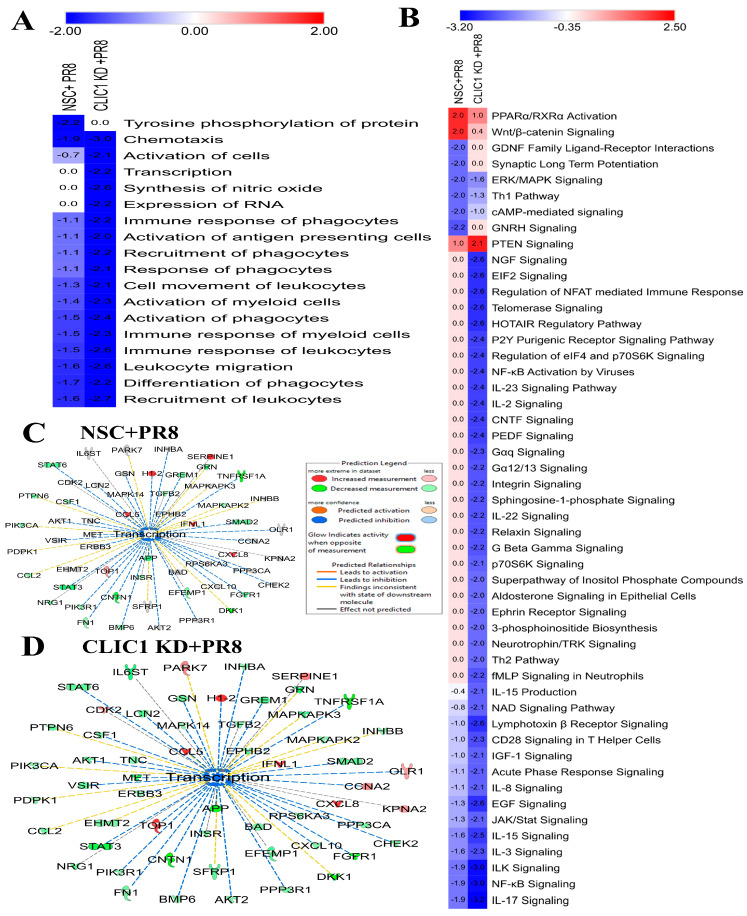
Impact of CLIC1 KD on cellular functions and signaling pathways in A549 during IAV infection. (**A**) Heatmap of cellular functions that were significantly dysregulated only by NSC or CLIC1 KD cells after IAV-PR8 infection. Numbers in the boxes indicate the activation/inactivation Z scores. (**B**) Heatmap of the signaling pathways significantly dysregulated only in NSC + PR8 or CLIC1 + PR8 cells. Numbers in the boxes indicate the activation/inactivation Z scores. Red: Activated; Blue: inhibited. (**C**) Impact of PR8 infection on cellular transcription process predicted by IPA analysis based on the significantly dysregulated proteins only in (**C**) NSC or (**D**) CLIC1 KD cells.

IPA also predicted CLIC1 KD might cause significant inhibition of immune cells (phagocytes, leukocytes, antigen-presenting cells, myeloid cells) activation and migration (Figure 6A). Eight signaling pathways (two activated, six inhibited) were significantly dysregulated during PR8 infection in A549 cells but were not impacted in CLIC1 KD cells after PR8 infection. However, 42 signaling pathways (one activated, forty-one inhibited) were significantly dysregulated in CLIC1 KD cells but were not significantly affected in wild-type cells after PR8 infection (Figure 6A). IL-17, NF-kB, EIF2, and NGF signaling were the top most affected pathways in CLIC1 KD cells but were not affected by PR8 infection in wild-type cells. PPARα/RXRα activation, Wnt/β-catenin signaling, GNRH signaling cAMP-mediated signaling, and ERK/MAPK signaling are the most prominent among the signaling pathways significantly affected by PR8 infection but were not impacted by the infection in CLIC1 KD cells (Figure 6B). Interestingly, cellular transcription processes were predicted to be significantly inhibited in CLIC1 KD cells after PR8 infection, based on the expression of 56 associated proteins, which was not impacted by PR8 infection in wild-type cells (Figure 6C,D). 

Bioinformatic analysis by IPA also predicted that 14 (10 activated, 4 inhibited) upstream regulators were significantly dysregulated in wild-type cells that were not impacted in CLIC1 KD cells. These upstream regulators are associated with phosphorylation of protein, migration of cells, transcription of RNA, transcription of DNA, apoptosis activation of leukocytes, and recruitment of cells (Supplementary Figure S1A,B). In contrast, PR8 infection in CLIC1 KD cells caused significant dysregulation of 37 (9 activated, 27 inhibited) upstream regulators that were not affected by PR8 infection in wild-type cells (Supplementary Figure S1A). These upstream regulators are involved in the regulation of different cellular functions, including necrosis, apoptosis, expression of RNA, transcription of DNA, transcription of RNA, cell viability, cell cycle progression, and migration of cells (Supplementary Figure S1C). 

## 4. Discussion

### 4.1. CLIC1 Knockdown Alters IAV-Mediated Host Proteomic Responses

By proteomic analysis, we found that the expression of several proteins was significantly altered in wild-type cells after IAV infection. For example, nascent polypeptide-associated complex subunit alpha (NACA), insulin-like growth factor-binding protein 6 (IGFBP6), peptidylprolyl isomerase F (PPIF), EPH receptor A3 (EPHA3), and interleukin 11 (IL11) were significantly up-regulated, and the expression of histone acetyltransferase 1 (HAT1), H2A.Z variant histone 1 (H2AZ1), peptidylprolyl isomerase D (PPID), calcium/calmodulin-dependent protein kinase II beta (CAMK2B), and aryl hydrocarbon receptor-interacting protein (API) were down-regulated but were not significantly affected in CLIC1 KD cells after PR8 infection. TGFB1 is the only protein that was significantly up-regulated in wild-type cells but was significantly down-regulated in CLIC1 KD cells after PR8 infection. TGFB1 is an essential protein for cell proliferation and differentiation, apoptosis, and inflammation [27]. Higher expression of TGFB1 has been associated with tumorigenesis [28]. Several viruses, including HBV, HCV, EBV, RSV, HPV, STLV-1, CMV, and HIV-1, can modulate the TGFB pathway during infection [29]. IAV infection also induces activation of the TGFB pathway, resulting in enhanced epithelial apoptosis, collagen deposition, and pulmonary fibrosis priming bacterial co-infection [30,31]. Interestingly, we found CLIC1 knockdown causes significant inhibition of TGFB1 expression. CLIC1 might be a critical protein for activation of the TGFB1 pathway during IAV infection. An in-depth study is necessary to understand the mechanism more clearly. 

In contrast, CLIC1 KD caused significant up-regulation of 2′-5′-oligoadenylate synthetase 1 (OAS1), cyclin dependent kinase 2 (CDK2), cyclin A2 (CCNA2), parkinsonism associated deglycase (PARK7), and down-regulation of MET proto-oncogene, receptor tyrosine kinase (MET), interleukin 6 cytokine family signal transducer (IL6ST), MAPK activated protein kinase 3 (MAPKAPK3), C-C motif chemokine ligand 13 (CCL13); these proteins were not significantly impacted by PR8 infection in wild-type cells (Figure 5B,C). 

NACA [32] and HAT1 [33] are potential host factors for Hepatitis B virus (HBV) replication. IGFBP6 can bind physically with the Orf virus (ORFV) ORFV024 protein, which can suppress the NF-kB signaling pathway and function as a critical regulator for early immune responses [34]. IL-11 is an anti-inflammatory factor, and its expression becomes elevated by virus infection and could be a potential target for novel antiviral development [35]. However, the role of EPHA3, H2AZ1, PPIF, PPID, PARK7, CCNA2, and MET in viral replication is not well known for any virus. CAMK2B is a critical host factor for influenza virus replication [36]. TGFBI regulates cell motility and transformation, although its involvement in viral replication is unknown [37]. OAS1 activates RNase L, which inhibits SARS-CoV-2 replication [38]. Interestingly CLIC1 KD also caused significantly higher expression of OAS1 protein in IAV-infected cells (Figure 5C). The replication of IAV could be inhibited by OAS1-induced RNase L-mediated pathways as it is for SARS-CoV-2, which is controlled by CLIC1 [38]. Further study is necessary to understand the interaction of OAS1 with CLIC1 and its function in IAV replication. CDK2 activates the viral DNA synthesis of herpes simplex virus 1 (HSV-1) and promotes HIV-1 and SARS-CoV-2 replication [39,40]. SARS-CoV-2 infection dysregulates CDK signaling pathways to arrest S/G2-like phase, creating favourable conditions for viral replication [41,42]. A higher expression of CDK2 might be another restriction factor that may have caused reduced replication of IAV in CLIC1 KD cells. IL-6 is a critical cytokine produced in response to infection or tissue damage and required to mount immune response sequentially [43,44,45]. A lower expression of IL-6 in CLIC1 KD cells may impair the appropriate activation of immune response in the cells. MAPKAPK3 is a critical host factor for chikungunya virus required for actin remodeling during replication [46]. However, the role of MAPKAPK3 in IAV replication is not well understood. 

### 4.2. CLIC1 Knockdown Cause Differential Regulation of Cellular Functions and Signaling Pathways Dysregulated by IAV Infection

Infection with low pathogenic IAV-H9N2 and high pathogenic H5N1 strains can cause cell death by necrosis [47,48]. However, IAV (H5N1)-NS1 protein can induce cell death by apoptosis [49,50]. Unlike previous observations, we found IAV-PR8 infection causes inhibition of apoptosis and necrosis-mediated cell death (Figure 5D). Interestingly, IAV-PR8 infection activated apoptosis and necrosis pathways in CLIC1 KD cells (Figure 5E), showing that CLIC1 may be involved in viral infection-mediated apoptosis and necrosis pathway activation.

IPA also predicted that CLIC1 KD could significantly reduce the activation of different immune cells and cellular transcription of DNA and RNA (Figure 6A,C; Supplementary Figure S1C) in IAV infected cells. Although cellular transcription was reduced, we detected a significantly higher number of viral RNA transcripts in the CLIC1 KD cells (Figure 4E), indicating that CLIC1 could be a critical protein for the cellular RNA and DNA transcription process; further investigation is required to delineate the mechanism. 

The PTEN signaling pathway was activated in CLIC1 KD cells but was not significantly activated in the wild-type cells after IAV-PR8 infection (Figure 6B). This pathway regulates the activation and differentiation of several immune cells and plays a critical role in maintaining immune homeostasis [51]. CLIC1 might be a regulator for suppression of PTEN signaling and IAV may induce overexpression of CLIC1 to inhibit activation of the pathway. Interestingly we found different interleukin signaling pathways (IL-17, IL-3, IL-15, IL-22, IL-2, IL-23, and IL-8) were significantly inhibited in the CLIC1 KD cells after IAV-PR8 infection. Interleukin signaling pathways are usually activated by most viral infections and play a central role in maintaining immune response [44,52,53,54,55]. However, previous studies have reported that IL-8 can promote Cytomegalovirus (CMV) replication by inhibition of the antiviral activity of Interferon-alpha (IFN-α) [56,57] and the expression of IL-8 was enhanced by IAV [58], respiratory syncytial virus [59], and rotavirus [60] infection. Thus, significant inhibition of the IL-8 signaling pathway in CLIC KD could be a cause for the reduction of IAV replication. 

### 4.3. CLIC1 Is Important for Later Stages of IAV Replication

In this study, we showed that CLIC1 KD significantly impacts the replication of IAV (Figure 2A), confirming the previous 96 well-based siRNA screening results [8]. However, we observed that CLIC1 KD had a greater impact on IAV strains pdm-09 and WSN than on PR8 (Figure 2D). PR8 and WSN are lab-adapted, mouse-passaged strains whereas pdm-09 is a human IAV strain. The dependency of viral replication may be different in lab-adapted strains compared to human IAV strains. In addition, treatment with at least 62.5 μM NPPB, a chloride channel inhibitor, also significantly reduced PR8 and N-Cal replication in A549 cells (Figure 3B,C), indicating CLIC1 is a critical host factor for the IAV life cycle. A previous study showed that treatment with 35.24 μM NPPB inhibited the entry of herpes simplex virus type 1 (HSV-1) into Vero cells [61]. However, varying concentrations of NPPB, ranging from 42 to 300 μM, were used on other cell lines, including primary cardiomyocytes [62] and human adenocarcinoma cells [63], to inhibit chloride channels. The differences in NPPB concentrations required to inhibit chloride channels might depend on the cell type and their respective responses to different viruses. In this study we also found that CLIC1 expression was significantly higher in IAV-infected cells (Figure 3A,B), as previously shown [7].

**Figure 7:**
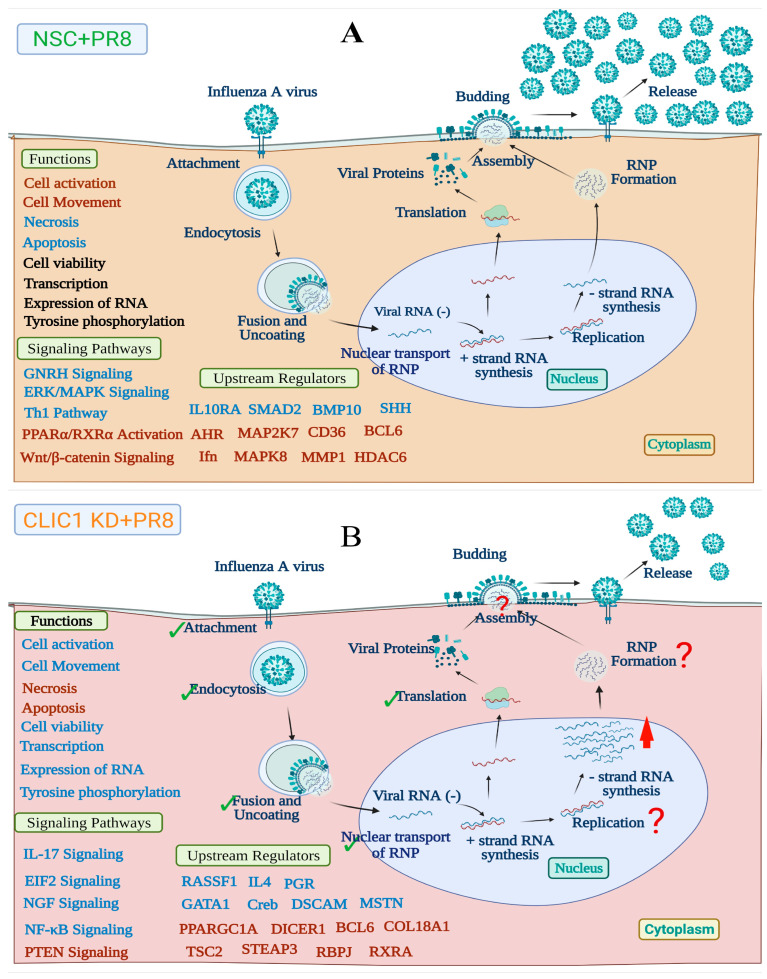
Proposed model showing the role of CLIC1 in IAV replication. (**A**) The replication cycle of IAV and effect of IAV infectionon cell function, signaling pathways and upstream regulators (**B**) Impact of CLIC1 KD on IAV replication and cellular function, signaling pathways and upstream regulators. Viral protein translation was not affected by CLIC1 KD, which indicates that earlier steps in IAV replication cycle, e.g., attachment, endocytosis, fusion, nuclear transport, and mRNA synthesis, should also be unaffected. A higher number of total viral RNAs were detected in the CLIC1 KD cells after PR8 infection, indicated by red arrow. However, we detected a significantly lower number of progeny viruses in the supernatant of CLIC1 KD cells compared to control. This suggests that CLIC1 is required in later stage(s) of viral replication. Activation and inhibition of cellular function, signaling pathways and upstream regulators were indicated by the front colors. Red: activated/up-regulated, Blue: suppressed/down-regulated and Black: unaffected. Green tick marks show the stages of IAV replication was not affected by CLIC1 knockdown The red question mark indicates the possible effect CLIC1 KD on stages of IAV replication.

Although CLIC1 KD reduced the number of progeny virus particles in the supernatant, the translation of viral proteins was not significantly affected in the CLIC1 KD cells during IAV infection (Figure 4A,C,D). This implies that earlier steps in the IAV replication cycle e.g., attachment, endocytosis, fusion, nuclear transport, and mRNA synthesis, were also unaffected in CLIC1 KD cells. Interestingly, a higher number of total viral RNA was detected in the CLIC1 KD cells after PR8 infection (Figure 7). This suggests that CLIC1 is required for later stage(s) of viral replication. Previous studies, based on RNAi screening, found that CLIC1 was a critical host factor for West Nile virus [18] and vaccinia virus [19]. However, the specific function(s) of CLIC1 in West Nile virus or Vaccinia virus replication is/are still unknown. However, several other host factors were also involved in the later stages of IAV replication including post-translational modification, RNA transcription, RNP formation, and assembly. Acidic nuclear phosphoprotein 32 family member A (ANP32A), which regulates p38 and Akt activity to modulate cell growth in some cancers [64], mediates assembly of the IAV replicase [65]. Aminoacyl tRNA synthase complex-interacting multifunctional protein 2 (AIMP2), required for assembly and stability of the aminoacyl-tRNA synthase complex [66], facilitates SUMOylation of IAV M1 proteins [67]. Cluster of differentiation 81 (CD81) is a transmembrane protein that mediates different signal transduction pathways to regulate cell development, activation, growth, and motility [68,69], involved in uncoating and budding of influenza virus [70]. ITCH is a ubiquitin ligase enzyme involved in immune responses, DNA repair, and cellular differentiation [71,72] and was found critical for influenza viral entry and uncoating processes [73]. The proline-glutamine rich splicing factor (SFPQ/PSF) is a nuclear protein involved in different cellular functions including cellular transcription, mRNA splicing [74], and it is required for influenza virus RNA transcription [68]. The associations of these proteins with CLIC1 are still unknown and need to be investigated in future studies.

## 5. Conclusions

In this study CLIC1 was identified as a critical host dependency factor essential for RNP production or IAV particle maturation. Furthermore, the chloride channel inhibitor NPPB had significant antiviral effects against multiple strains of IAV. More research is needed to investigate the precise mechanism of CLIC1 in IAV replication using CLIC1 knockout (KO) cell lines or mice models to understand the therapeutic importance of this protein as a target for anti-IAV drug development. However, in this study we only tested the role of CLIC1 on IAV replication, although CLIC proteins are highly homologous and have similar functions. The role of other CLIC proteins in virus replication and pathogenesis needs to be investigated in future studies. 

## Figures and Tables

**Table 1:** Numbers of significantly dysregulated proteins in wild type and CLIC1 KD cells after PR8 infection.

Range of Fold Change	PR8 (Protein No.)	Total Significant (Protein No.)	CLIC1 KD +PR8 (Protein No.)	Total Significant (Protein No.)
and F.C. > 1.00	76	218	213	352
and F.C. < 1.00	142		139	
and F.C. > 1.10	68	197	88	279
and F.C. < −1.10	129		191	
and F.C. > 1.20	43	144	31	183
and F.C. < −1.20	101		152	
and F.C. > 1.30	31	116	22	149
and F.C. < −1.30	85		127	
and F.C. > 1.50	20	68	18	94
and F.C. < −1.50	48		76	
and F.C. > 1.60	17	57	16	75
and F.C. < −1.60	40		59	
and F.C. > 2.00	11	33	6	31
and F.C. < −2.00	22		25	
and F.C. > 2.50	8	22	6	27
and F.C. < −2.50	14		21	

Significance was determined by *T*-test and Z-score as detailed in Section 2 from three biological replicates. The list of proteins dysregulated ≥ 1.5-fold in either direction is listed in Table 2.

**Table 2:** List of significantly dysregulated proteins in wild type and CLIC1 KD cells after PR8 infection.

Type(s)	Symbols	Entrez Gene Name	NSC + PR8 (FC)	*p*-Value	CLIC1 KD + PR8 (FC)	*p*-Value	Location
Cytokines	CXCL8	C-X-C motif chemokine ligand 8	10.27	2.21 × 10^−5^	5.74	1.97 × 10^−4^	Extracellular Space
	CCL5	C-C motif chemokine ligand 5	6.39	3.59 × 10^−3^	9.88	1.05 × 10^−3^	Extracellular Space
	IFNL1	interferon lambda 1	2.87	3.46 × 10^−2^	4.36	6.17 × 10^−3^	Extracellular Space
	LTA	lymphotoxin alpha	−1.50	2.96 × 10^−3^	−1.53	1.45 × 10^−2^	Extracellular Space
	IL17D	interleukin 17D	−1.43	2.76 × 10^−2^	−1.52	1.70 × 10^−3^	Extracellular Space
	CCL2	C-C motif chemokine ligand 2	−1.40	4.31 × 10^−2^	−1.54	5.80 × 10^−3^	Extracellular Space
	CCL13	C-C motif chemokine ligand 13	−1.42	9.43 × 10^−2^	−1.82	4.77 × 10^−2^	Extracellular Space
Enzymes	PPIF	peptidylprolyl isomerase F	1.73	2.93 × 10^−2^	1.04	5.15 × 10^−1^	Cytoplasm
	TOP1	DNA topoisomerase I	1.50	1.82 × 10^−3^	2.99	4.83 × 10^−2^	Nucleus
	EFEMP1	EGF containing fibulin extracellular matrix protein 1	−1.50	1.75 × 10^−2^	−1.71	5.69 × 10^−3^	Extracellular Space
	PPID	peptidylprolyl isomerase D	−1.53	1.02 × 10^−2^	−1.15	4.87 × 10^−1^	Cytoplasm
	AKR1A1	aldo-keto reductase family 1 member A1	−1.72	3.82 × 10^−2^	−1.54	3.50 × 10^−2^	Cytoplasm
	HAT1	histone acetyltransferase 1	−2.37	2.10 × 10^−4^	−1.34	1.69 × 10^−1^	Nucleus
	CNTN1	contactin 1	−2.93	1.58 × 10^−2^	−4.07	9.07 × 10^−4^	Plasma Membrane
	OAS1	2′-5′-oligoadenylate synthetase 1	1.28	1.53 × 10^−1^	1.95	9.62 × 10^−3^	Cytoplasm
	PARK7	Parkinsonism associated deglycase	1.10	4.97 × 10^−1^	1.72	3.38 × 10^−2^	Nucleus
	FN1	fibronectin 1	−1.32	6.79 × 10^−3^	−1.50	8.95 × 10^−3^	Extracellular Space
	CHST15	carbohydrate sulfotransferase 15	−1.40	1.78 × 10^−1^	−1.51	3.64 × 10^−2^	Plasma Membrane
	CA7	carbonic anhydrase 7	−1.40	3.24 × 10^−2^	−1.52	7.96 × 10^−3^	Cytoplasm
	ENTPD5	ectonucleoside triphosphate diphosphohydrolase 5 (inactive)	−1.49	4.88 × 10^−3^	−1.61	4.17 × 10^−3^	Cytoplasm
	GNS	glucosamine (N-acetyl)−6-sulfatase	−1.22	7.69 × 10^−2^	−1.68	3.01 × 10^−3^	Cytoplasm
	RNASEH1	ribonuclease H1	−1.13	5.80 × 10^−1^	−1.74	4.10 × 10^−2^	Nucleus
	TYMS	thymidylate synthetase	−1.13	1.21 × 10^−1^	−1.82	2.42 × 10^−3^	Nucleus
	GPNMB	glycoprotein nmb	−1.40	5.17 × 10^−3^	−2.20	3.57 × 10^−3^	Plasma Membrane
Growth factors	BMP6	bone morphogenetic protein 6	−1.56	3.23 × 10^−2^	−1.42	4.85 × 10^−2^	Extracellular Space
	NRG1	neuregulin 1	−1.61	4.73 × 10^−2^	−1.63	8.98 × 10^−3^	Plasma Membrane
	FGF6	fibroblast growth factor 6	−1.63	5.64 × 10^−3^	−1.65	3.08 × 10^−3^	Extracellular Space
	GRN	granulin precursor	−2.09	4.60 × 10^−3^	−2.51	5.74 × 10^−3^	Extracellular Space
	DKK1	dickkopf WNT signaling pathway inhibitor 1	−3.61	5.46 × 10^−3^	−5.88	9.57 × 10^−5^	Extracellular Space
Kinases	EPHA2	EPH receptor A2	3.00	4.71 × 10^−3^	1.74	2.53 × 10^−2^	Plasma Membrane
	STC1	stanniocalcin 1	2.40	2.88 × 10^−3^	1.54	1.69 × 10^−3^	Extracellular Space
	EPHA3	EPH receptor A3	1.60	2.03 × 10^−2^	1.21	7.23 × 10^−2^	Plasma Membrane
	NAGK	N-acetylglucosamine kinase	−1.50	3.50 × 10^−2^	−1.30	7.65 × 10^−2^	Cytoplasm
	CAMK2D	calcium/calmodulin dependent protein kinase II delta	−1.50	4.20 × 10^−2^	−1.24	4.63 × 10^−2^	Cytoplasm
	CAMK2B	calcium/calmodulin dependent protein kinase II beta	−1.55	5.96 × 10^−3^	−1.31	5.47 × 10^−2^	Cytoplasm
	PRKCG	protein kinase C gamma	−1.61	1.95 × 10^−2^	−1.72	6.67 × 10^−3^	Cytoplasm
	PIK3CA	phosphatidylinositol-4,5-bisphosphate 3-kinase catalytic subunit alpha	−1.63	4.25 × 10^−2^	−1.39	1.87 × 10^−2^	Cytoplasm
	PIK3R1	phosphoinositide-3-kinase regulatory subunit 1	−1.63	4.25 × 10^−2^	−1.39	1.87 × 10^−2^	Cytoplasm
	EFNA2	ephrin A2	−1.66	4.84 × 10^−3^	−1.86	5.30 × 10^−4^	Plasma Membrane
	FGFR1	fibroblast growth factor receptor 1	−2.07	3.29 × 10^−3^	−3.77	1.83 × 10^−3^	Plasma Membrane
	CDK2	cyclin dependent kinase 2	−1.26	2.15 × 10^−1^	1.65	3.12 × 10^−02^	Nucleus
	RPS6KA3	ribosomal protein S6 kinase A3	−1.53	6.31 × 10^−2^	−1.54	4.80 × 10^−02^	Cytoplasm
	ERBB3	erb-b2 receptor tyrosine kinase 3	−1.49	8.60 × 10^−2^	−1.55	4.50 × 10^−03^	Plasma Membrane
	CHEK2	checkpoint kinase 2	−1.35	5.81 × 10^−2^	−1.62	1.00 × 10^−02^	Nucleus
	AKT1	AKT serine/threonine kinase 1	−1.37	1.55 × 10^−1^	−1.69	1.96 × 10^−2^	Cytoplasm
	AKT3	AKT serine/threonine kinase 3	−1.37	1.55 × 10^−1^	−1.69	1.96 × 10^−2^	Cytoplasm
	INSR	insulin receptor	−1.43	2.23 × 10^−2^	−1.69	4.86 × 10^−2^	Plasma Membrane
	EPHB2	EPH receptor B2	−1.08	2.10 × 10^−1^	−1.71	4.86 × 10^−3^	Plasma Membrane
	MAPKAPK3	MAPK activated protein kinase 3	−1.67	6.14 × 10^−2^	−1.90	1.07 × 10^−2^	Nucleus
	MET	MET proto-oncogene, receptor tyrosine kinase	−1.50	9.39 × 10^−2^	−2.96	4.18 × 10^−4^	Plasma Membrane
Peptidase	CTSA	cathepsin A	−2.11	4.83 × 10^−3^	−3.02	2.56 × 10^−3^	Cytoplasm
	C1R	complement C1r	1.51	2.00 × 10^−1^	1.62	4.90 × 10^−2^	Extracellular Space
	ADAMTS1	ADAM metallopeptidase with thrombospondin type 1 motif 1	−1.37	9.97 × 10^−3^	−1.50	2.30 × 10^−2^	Extracellular Space
	PCSK9	proprotein convertase subtilisin/kexin type 9	−5.21	7.16 × 10^−3^	−5.05	1.15 × 10^−4^	Extracellular Space
Phosphatase	PTPN6	protein tyrosine phosphatase non-receptor type 6	−1.54	1.17 × 10^−3^	−1.55	6.93 × 10^−3^	Cytoplasm
Transcription regulator	NACA	nascent polypeptide associated complex subunit alpha	2.11	1.80 × 10^−2^	1.40	9.21 × 10^−2^	Cytoplasm
	HMGB1	high mobility group box 1	−1.51	1.38 × 10^−2^	−1.09	6.00 × 10^−1^	Nucleus
	AIP	aryl hydrocarbon receptor interacting protein	−1.55	1.13 × 10^−2^	−1.29	1.48 × 10^−1^	Nucleus
	SMAD2	SMAD family member 2	−1.62	3.48 × 10^−2^	−1.56	1.27 × 10^−2^	Nucleus
	STAT6	signal transducer and activator of transcription 6	−1.66	2.05 × 10^−2^	−1.55	2.17 × 10^−2^	Nucleus
	STAT3	signal transducer and activator of transcription 3	−2.09	7.86 × 10^−3^	−2.78	3.71 × 10^−3^	Nucleus
	EIF4EBP2	eukaryotic translation initiation factor 4E binding protein 2	−1.44	3.52 × 10^−2^	−1.53	7.05 × 10^−3^	Cytoplasm
Transmembrane receptor	TNFRSF10D	TNF receptor superfamily member 10d	3.63	1.02 × 10^−2^	1.82	3.01 × 10^−4^	Plasma Membrane
	B2M	beta-2-microglobulin	2.49	1.30 × 10^−4^	1.55	2.11 × 10^−2^	Plasma Membrane
	PLAUR	plasminogen activator, urokinase receptor	1.87	8.83 × 10^−3^	1.60	2.69 × 10^−3^	Plasma Membrane
	KIR2DL4	killer cell immunoglobulin like receptor, two Ig domains and long cytoplasmic tail 4	−1.70	1.01 × 10^−2^	−1.85	1.94 × 10^−3^	Plasma Membrane
	MICB	MHC class I polypeptide-related sequence B	−1.74	3.03 × 10^−2^	−2.85	8.76 × 10^−4^	Plasma Membrane
	GFRA1	GDNF family receptor alpha 1	−1.95	1.53 × 10^−2^	−1.89	2.41 × 10^−3^	Plasma Membrane
	NRP1	neuropilin 1	−2.15	1.80 × 10^−2^	−3.11	2.71 × 10^−4^	Plasma Membrane
	TNFRSF21	TNF receptor superfamily member 21	−2.52	8.55 × 10^−4^	−3.49	8.35 × 10^−4^	Plasma Membrane
	RTN4R	reticulon 4 receptor	−2.98	1.40 × 10^−2^	−2.77	2.31 × 10^−4^	Plasma Membrane
	TNFRSF1A	TNF receptor superfamily member 1A	−3.92	5.43 × 10^−3^	−3.45	2.82 × 10^−3^	Plasma Membrane
	PGLYRP1	peptidoglycan recognition protein 1	−1.47	5.55 × 10^−2^	−1.52	2.06 × 10^−2^	Plasma Membrane
	SFRP1	secreted frizzled related protein 1	−1.21	6.59 × 10^−2^	−1.61	9.71 × 10^−3^	Plasma Membrane
	RELT	RELT TNF receptor	−1.49	2.12 × 10^−2^	−1.61	1.26 × 10^−2^	Plasma Membrane
	PLXNB2	plexin B2	−1.33	3.82 × 10^−2^	−1.73	4.14 × 10^−3^	Plasma Membrane
	IL6ST	interleukin 6 cytokine family signal transducer	−1.21	5.44 × 10^−3^	−2.01	3.57 × 10^−3^	Plasma Membrane
Transporter	ATP5PO	ATP synthase peripheral stalk subunit OSCP	1.59	2.67 × 10^−02^	1.76	1.59 × 10^−2^	Cytoplasm
	BPI	bactericidal permeability increasing protein	−1.61	4.50 × 10^−03^	−1.82	3.75 × 10^−3^	Plasma Membrane
	SNX4	sorting nexin 4	−1.61	2.04 × 10^−02^	−1.90	4.02 × 10^−2^	Cytoplasm
	LBP	lipopolysaccharide binding protein	−1.42	1.72 × 10^−1^	−1.68	1.66 × 10^−3^	Plasma Membrane
	MCL1	MCL1 apoptosis regulator, BCL2 family member	−1.40	4.72 × 10^−2^	−1.69	1.97 × 10^−3^	Cytoplasm
	LCN2	lipocalin 2	−1.23	1.24 × 10^−1^	−1.70	1.24 × 10^−3^	Extracellular Space
Other	ISG15	ISG15 ubiquitin like modifier	7.21	4.52 × 10^−3^	9.38	6.99 × 10^−4^	Extracellular Space
	SERPINE1	serpin family E member 1	5.98	3.82 × 10^−3^	1.85	9.03 × 10^−3^	Extracellular Space
	H1-2	H1.2 linker histone, cluster member	2.77	7.98 × 10^−3^	6.59	1.82 × 10^−2^	Nucleus
	CST3	cystatin C	1.97	3.32 × 10^−3^	1.42	3.29 × 10^−2^	Extracellular Space
	IGFBP6	insulin like growth factor binding protein 6	1.96	5.31 × 10^−4^	1.24	1.91 × 10^−2^	Extracellular Space
	IGFBP2	insulin like growth factor binding protein 2	1.81	2.12 × 10^−2^	1.17	6.36 × 10^−3^	Extracellular Space
	CD55	CD55 molecule (Cromer blood group)	1.59	5.56 × 10^−4^	1.17	2.10 × 10^−1^	Plasma Membrane
	TGFBI	transforming growth factor beta induced	1.51	1.41 × 10^−2^	−1.35	1.08 × 10^−3^	Extracellular Space
	GREM1	gremlin 1, DAN family BMP antagonist	−1.54	1.05 × 10^−2^	−1.67	9.10 × 10^−3^	Extracellular Space
	SLITRK5	SLIT and NTRK like family member 5	−1.56	1.40 × 10^−2^	−1.40	2.51 × 10^−2^	Plasma Membrane
	RSPO2	R-spondin 2	−1.65	1.75 × 10^−2^	−1.86	5.72 × 10^−3^	Extracellular Space
	MICA	MHC class I polypeptide-related sequence A	−1.77	4.92 × 10^−2^	−2.79	8.23 × 10^−3^	Plasma Membrane
	CFH	complement factor H	−1.84	5.70 × 10^−3^	−1.87	8.71 × 10^−3^	Extracellular Space
	APP	amyloid beta precursor protein	−1.88	1.78 × 10^−2^	−3.22	2.95 × 10^−3^	Plasma Membrane
	MFGE8	milk fat globule EGF and factor V/VIII domain containing	−2.01	4.16 × 10^−2^	−2.02	4.08 × 10^−3^	Extracellular Space
	H2AZ1	H2A.Z variant histone 1	−2.08	1.21 × 10^−2^	−1.84	6.41 × 10^−2^	Nucleus
	AMIGO2	adhesion molecule with Ig like domain 2	−2.62	1.93 × 10^−2^	−2.54	7.72 × 10^−3^	Plasma Membrane
	KIF23	kinesin family member 23	−2.63	8.74 × 10^−3^	−1.68	7.04 × 10^−3^	Cytoplasm
	SERPINE2	serpin family E member 2	−2.65	1.65 × 10^−4^	−2.29	2.28 × 10^−4^	Extracellular Space
	DKK4	dickkopf WNT signaling pathway inhibitor 4	−3.62	8.22 × 10^−3^	−4.96	9.37 × 10^−5^	Extracellular Space
	UNC5D	unc-5 netrin receptor D	−4.32	9.53 × 10^−4^	−4.81	2.97 × 10^−4^	Plasma Membrane
	LAMA1	laminin subunit alpha 1	−4.38	1.17 × 10^−3^	−6.45	7.32 × 10^−4^	Extracellular Space
	LAMB1	laminin subunit beta 1	−4.38	1.17 × 10^−3^	−6.45	7.32 × 10^−4^	Extracellular Space
	LAMC1	laminin subunit gamma 1	−4.38	1.17 × 10^−3^	−6.45	7.32 × 10^−4^	Extracellular Space
	CCNA2	cyclin A2	−1.26	2.15 × 10^−1^	1.65	3.12 × 10^−2^	Nucleus
	GSN	gelsolin	−1.61	1.79 × 10^−1^	−1.50	4.78 × 10^−2^	Extracellular Space
	COLEC11	collectin subfamily member 11	−1.42	2.68 × 10^−2^	−1.51	1.63 × 10^−2^	Extracellular Space
	VSIR	V-set immunoregulatory receptor	−1.27	2.37 × 10^−2^	−1.56	3.04 × 10^−3^	Plasma Membrane
	HSPB1	heat shock protein family B (small) member 1	−1.05	5.86 × 10^−1^	−1.57	5.91 × 10^−4^	Cytoplasm
	BGN	biglycan	−1.39	1.10 × 10^−2^	−1.59	2.28 × 10^−2^	Extracellular Space
	LGALS8	galectin 8	−1.57	6.70 × 10^−2^	−1.63	7.61 × 10^−3^	Extracellular Space
	TIMP2	TIMP metallopeptidase inhibitor 2	−1.03	6.93 × 10^−1^	−1.69	3.88 × 10^−3^	Extracellular Space
	LRIG3	leucine rich repeats and immunoglobulin like domains 3	−1.19	5.15 × 10^−2^	−1.74	1.62 × 10^−2^	Extracellular Space
	NTN4	netrin 4	−1.47	8.19 × 10^−3^	−1.74	7.80 × 10^−4^	Extracellular Space

List of proteins with fold change up-regulated ≥ 1.5 (orange) or down-regulated ≤ −1.5 (Blue) FC = fold change. red: *p*-value < 0.05. In our previous study, we used the SOMAScan data for NSC + PR8 for a different analysis [8].

## Data Availability

Data are contained within the article and supplementary materials.

## Supplementary Materials

The following supporting information can be downloaded at: https://www.mdpi.com/article/10.3390/v16010129/s1, Supplementary Figure S1. Impact of CLIC1 KD on upstream regulators in A549 during IAV infection. (A) Heatmap of upstream regulators that were significantly dysregulated only by NSC or CLIC1 KD cells after IAV-PR8 infection. Numbers in the box indicates the activation/ inactivation Z score. Red: Activated; Blue: inhibited. (B) Association of the upstream regulators dysregulated only in NSC + PR8 cells with cellular functions. (C) Association of the upstream regulators dysregulated only in CLIC1 + PR8 cells with cellular functions. Supplementary Figure S2. Validation of SOMAscan results by Western blot. A549 cells were treated either with scrambled siRNA (NSC) or CLIC1 siRNA (CLIC1 KD) and infected with Influenza A virus (PR8; +) or Mock infected as control (−). Cell lysates were collected at 24 h post infection. (A) Western blot was done to validate the expression of cellular proteins STAT1, STAT3, PSMA2 and CLIC1. Viral NS1 protein expression was determined to confirm viral infection. (B). Expression value of the proteins from Western-blot were quantified from three replicates and plotted side-by-side with SOMAscan expression values for comparison. NSC = Scrambled siRNA control, WB = Western blot, KD = Knockdown.
