# Supplementary material for: Chloride Intracellular Channel Protein 1 (CLIC1) Is a Critical Host Cellular Factor for Influenza A Virus Replication
Source: Viruses. 2024 Jan 16;16(1):129. doi: 10.3390/v16010129 (PMC10819074; doi:10.3390/v16010129)
Supplement: Supplementary file 1 [file viruses-16-00129-s001.zip › viruses-2765556-supplementary.pdf]

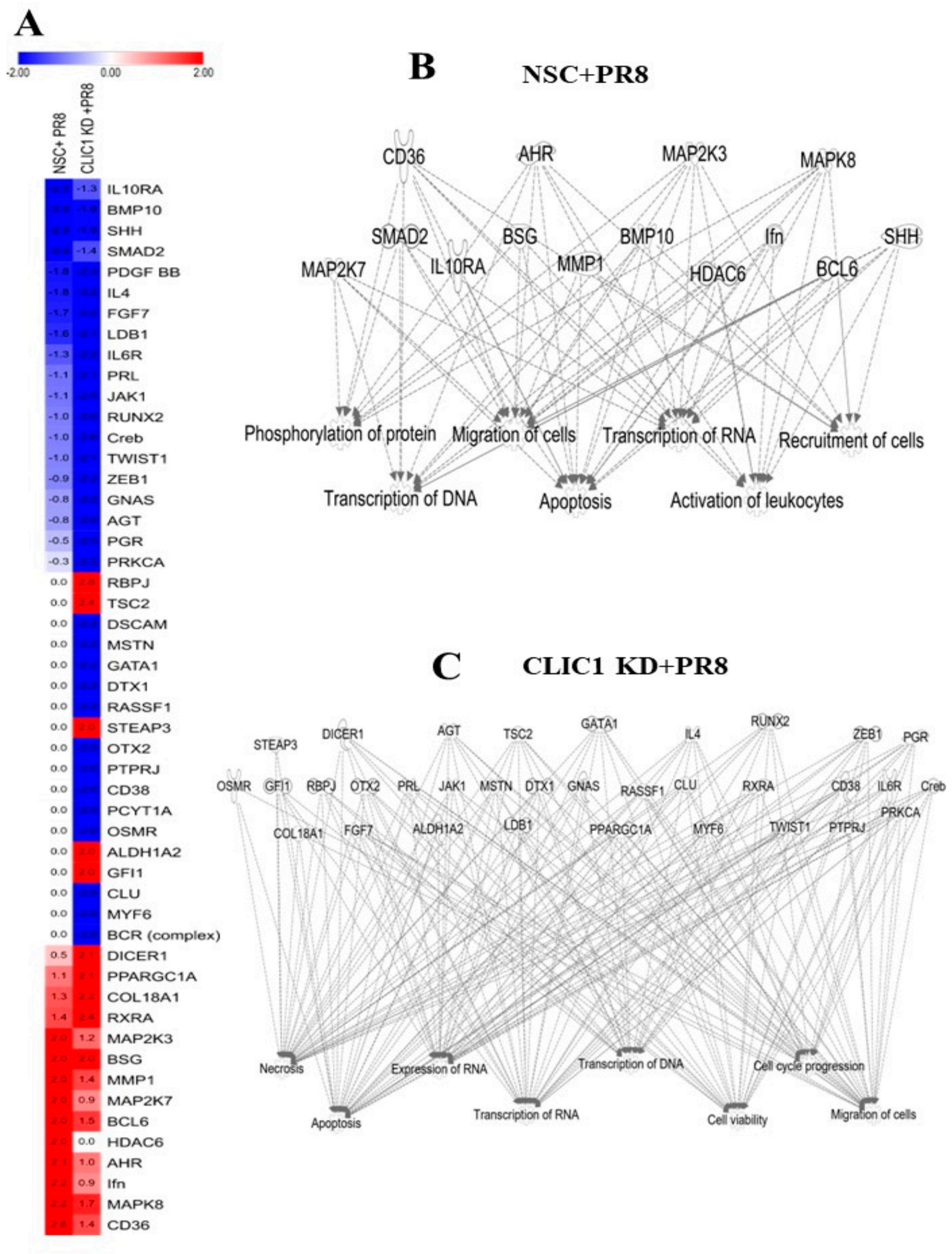

Supplementary Figure S1. Impact of CLIC1 KD on upstream regulators in A549 during IAV infection. A. Heatmap of upstream regulators that were significantly dysregulated only by NSC or CLIC1 KD cells after IAV-PR8 infection. Numbers in the boxes indicate the activation/inactivation Z score. Red: activated; Blue: inhibited. **B.** Association of the upstream regulators dysregulated only in NSC+PR8 cells with cellular functions. **C.** Association of the upstream regulators dysregulated only in CLIC1+PR8 cells with cellular functions.

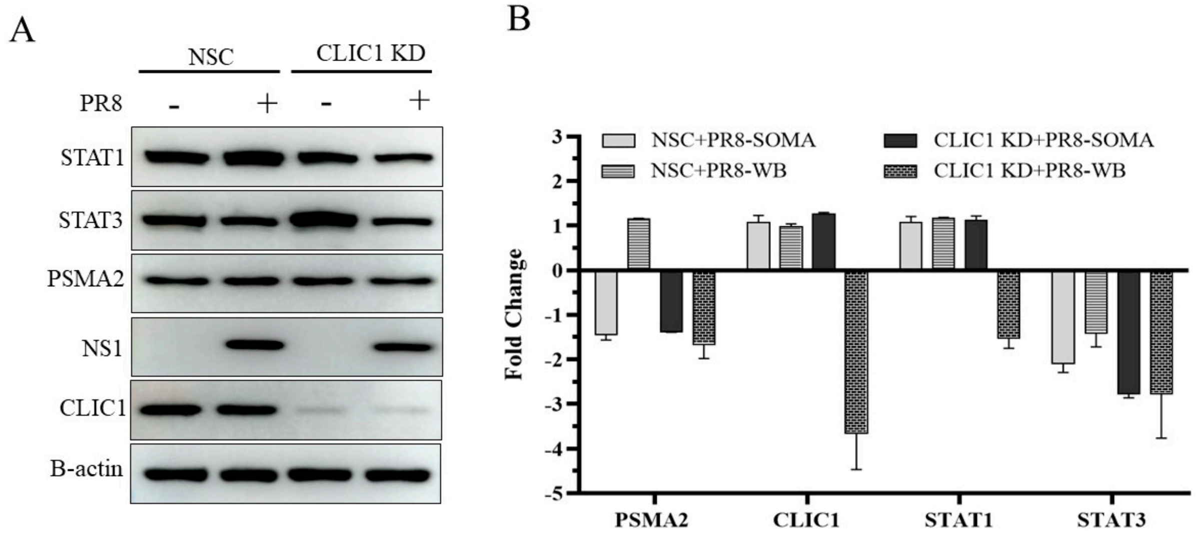

Supplementary Figure S2. Validation of SOMAscan results by Western blot. A549 cells were treated either with scrambled siRNA (NSC) or CLIC1 siRNA (CLIC1 KD) and infected with Influenza A virus (PR8; +) or Mock infected as control (-). Cell lysates were collected at 24 hours post infection. **A.** Western blot was done to validate the expression of cellular proteins STAT1, STAT3, PSMA2 and CLIC1. Viral NS1 protein expression was determined to confirm viral infection. **B**. Expression value of the proteins from Western-blot were quantified from three replicates and plotted side-by-side with SOMAscan expression values for comparison. NSC = Scrambled siRNA control, WB = Western blot, KD = Knockdown.
